# Novel Multivariable Evolutionary Algorithm-Based Method for Modal Reconstruction of the Corneal Surface from Sparse and Incomplete Point Clouds

**DOI:** 10.3390/bioengineering10080989

**Published:** 2023-08-21

**Authors:** Francisco L. Sáez-Gutiérrez, Jose S. Velázquez, Jorge L. Alió del Barrio, Jorge L. Alio, Francisco Cavas

**Affiliations:** 1 Department of Structures, Construction and Graphical Expression, Technical University of Cartagena, 30202 Cartagena, Spain; francisco.saez@upct.es (F.L.S.-G.); jose.velazquez@upct.es (J.S.V.); 2 Division of Ophthalmology, Miguel Hernández University, 03690 Alicante, Spain; jorge_alio@hotmail.com (J.L.A.d.B.); jlalio@vissum.com (J.L.A.); 3 Keratoconus Unit of Vissum Corporation Alicante, 03690 Alicante, Spain; 4 Department of Refractive Surgery, Vissum Corporation Alicante, 03690 Alicante, Spain

**Keywords:** genetic algorithm, corneal surface reconstruction, computer-aided design

## Abstract

Three-dimensional reconstruction of the corneal surface provides a powerful tool for managing corneal diseases. This study proposes a novel method for reconstructing the corneal surface from elevation point clouds, using modal schemes capable of reproducing corneal shapes using surface polynomial functions. The multivariable polynomial fitting was performed using a non-dominated sorting multivariable genetic algorithm (NS-MVGA). Standard reconstruction methods using least-squares discrete fitting (LSQ) and sequential quadratic programming (SQP) were compared with the evolutionary algorithm-based approach. The study included 270 corneal surfaces of 135 eyes of 102 patients (ages 11–63) sorted in two groups: control (66 eyes of 33 patients) and keratoconus (KC) (69 eyes of 69 patients). Tomographic information (Sirius, Costruzione Strumenti Oftalmici, Italy) was processed using Matlab. The goodness of fit for each method was evaluated using mean squared error (MSE), measured at the same nodes where the elevation data were collected. Polynomial fitting based on NS-MVGA improves MSE values by 86% compared to LSQ-based methods in healthy patients. Moreover, this new method improves aberrated surface reconstruction by an average value of 56% if compared with LSQ-based methods in keratoconus patients. Finally, significant improvements were also found in morpho-geometric parameters, such as asphericity and corneal curvature radii.

## 1. Introduction

In ophthalmological clinical practice, the morpho-geometric analysis of corneal structure allows for obtaining indices to evaluate corneal irregularities that affect patients’ visual quality [1,2,3]. These irregularities are generally present in corneal ectasias, and more specifically in keratoconus pathology, which is one of the most studied diseases of the human eye’s anterior segment [4,5,6]. In the scientific literature, several studies have used different morphological characterization techniques of the cornea [7,8,9,10,11]. However, a geometric reconstruction that aspires to be integral, patient-specific, and more deterministic than stochastic could only be feasible through using a computationally viable approach that reaches high accuracy and sensitivity in its reconstruction, even in the presence of complex corneal surface data dispersion and/or the absence of some geometrical information.

This data dispersion or absence can occur during the tomographer’s measurement acquisition process due to errors, which can be of a double nature: the so-called extrinsic errors, such as those caused by instability in the tear film [12], or the intrinsic errors, such as those due to noise present during the measurement acquisition process [12].

Regarding corneal reconstruction methods, there are two well-established paths: modal and zonal methods. Modal methods are based on the approximation of the surface through a combination of basic functions (modes) defined globally throughout the data domain, which may depend on a certain number of parameters and the necessary amount of them to recover the relevant information of the surface, trying to avoid over-fitting their measurement error [13]. Zonal methods are based on dividing the data domain into more elementary subdomains and approximating the surface in each defined subdomain independently of the rest [13,14].

In clinical practice, modal methods used are based on Zernike polynomials, which have shown high accuracy in slightly deformed corneas and good robustness against noise present in equipment during the measurement acquisition process [3], which gives them a lower dependence on measurement acquisition errors [14]. However, these polynomials have some problems due to their global nature, that is, in corneas that present significant surface irregularities, such as in the case of advanced keratoconus; these polynomials require high orders to perform a reliable reconstruction of corneal geometry, for which they use fitting tools such as least squares (LSQ) [15], or sequential quadratic programming (SQP) [16], but both generate instabilities against local minima caused by the discontinuities as mentioned above [17,18,19]. Therefore, it would be of interest to develop a modal reconstruction procedure that is not only accurate when irregular surfaces are present but also computationally viable in clinical practice.

Genetic algorithms (GA) are modal reconstruction methods not affected by local minimum fitting, which are characterized by using a search space consisting of complete solutions. Genetic algorithms have been successfully used in different fields to reconstruct complex geometric surfaces [20,21,22].

This study establishes a new modal method for corneal surface reconstruction using genetic algorithm fitting. Specifically, this research aims to analyze and compare the results of this fitting method to other methods by evaluating corneal morpho-geometric parameters, such as curvature radius or asphericity, in different clinical scenarios.

## 2. Materials and Methods

**Figure 1:**
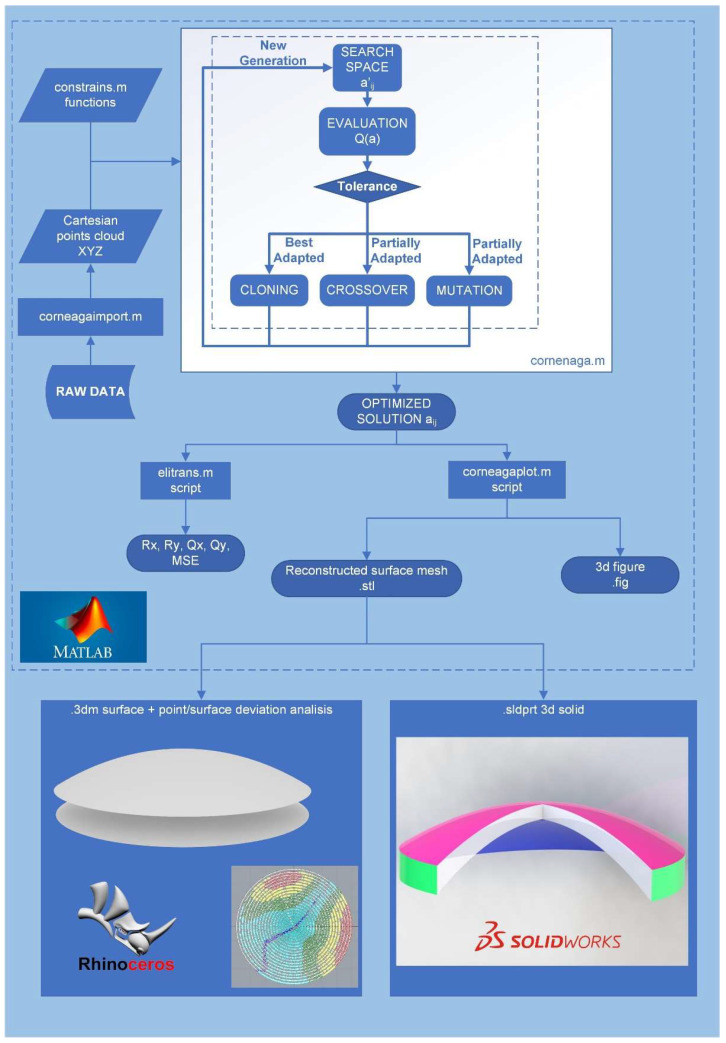
Global flowchart of the method. Rx, Ry: Radii of curvature of the corneal surface in x and y axes. Qx, Qy: asphericity of the corneal surface in x and y axes, MSE: mean squared error.

The corneal model reconstruction method proposed in this work is shown in Figure 1. Briefly, its procedure can be explained as follows: source data from the optical tomography are entered, after data conversion, into a script called “*corneaga.m*” run under Matlab software. The script uses *ga* function of the genetic algorithm to obtain the optimal parameters of the modal fit of the corneal surface to an implicit equation. Once the reconstruction parameters are obtained, the surface is graphically represented within the Matlab environment and can then be exported as a mesh. Additionally, the morpho-geometric parameters of the reconstructed cornea and the goodness of fit are obtained using their mean squared error (MSE). The obtained surface can be graphically represented and analyzed using CAD software such as Rhinoceros (version 7.0; McNeil Inc., NJ, USA) or Solidworks (version 2022; Dassault Systèmes, Vélizy-Villacoublay, France). In Figure 1 below, all the executed phases are shown in detail.

### 2.1. Data Source

This experimental study included ophthalmological data from 102 patients aged between 11 and 63. In order to test the method on cases of keratoconus (KC), the sample was divided into four groups according to the Amsler–Krumeich (AK) keratoconus scale [23]: 66 healthy eyes (control group: 33 patients, both eyes), 27 eyes grade 1, 26 eyes grade 2, and 16 eyes of grade 3 or higher. Only one eye of each subject was selected to avoid correlation between each pair of eyes in KC groups. All data used in the study were obtained from the IBERIA BIOBANK database (Universidad Miguel Hernández de Elche, OFTARED-Instituto de Salud Carlos III), which contains more than 500 keratoconus eyes at the moment, classified according to different scales and criteria. The databank was created in the framework of the National Network of Ophthalmological Research, also known as OFTARED in Spain. 

Also, the study was approved by the UPCT ethics committee (CEI21_001), following the ethical standards of the Helsinki Declaration (7th revision, October 2013, Fortaleza, Brazil). For each group of eyes, the inclusion criteria were: for the control group, healthy patients with no prior history of ocular pathologies; and, for the pathological groups, patients with diagnosed keratoconus classified according to the Amsler–Krumeich scale [24,25].

### 2.2. Data Acquisition

In order to collect the experimental data, each eye underwent a clinical examination with Scheimpflug tomography (OT) using the Sirius system (Costruzione Strumenti Oftalmici, Italy). A single experienced doctor performed all tests. A minimum of three corneal tomographies were performed for each eye, and the best one was selected according to the quality of instrument acquisition. After that, all corneal tomography data were exported in a .csv data file to be processed by the modal reconstruction algorithm, studied through morpho-geometric characterization, and validated by our research group [26].

### 2.3. NS-MVGA-Based Modal Reconstruction 

The surface reconstruction of the cornea proposed in this research is based on the following phases:

#### 2.3.1. Discretization

The tomographic data file, obtained from the Sirius tomographer, comprises elevation data expressed as spatial points. Data are obtained as discrete points P_s_, s = 1, …, 6144, in polar coordinates (phi, theta), distributed in radii phi_i_ = i/24, i = 1, …, 24, and semi meridians teta_j_, j = 1, …, 256 between 0 and 2 pi. Using modal functions in Cartesian form requires input data in three-dimensional Cartesian point format XYZ [27]. The spatial points in polar format were transformed into Cartesian format through a script programmed in Matlab. The script, called “*corneagaimport.m*”, performs two processes: (i) it obtains the Cartesian coordinates of the points that are in a polar format in the source data file by transforming each polar point [i,j] into its corresponding triangulation [i0.2,j360/256u] in radius and angle taking radii every 0.2 mm and 256 points per radius, and, subsequently, (ii) it eliminates invalid data in the experimental acquisition process (value = −1000).

#### 2.3.2. Fitting of the Modal Surface Function

In the next step, a modal fitting algorithm of the surface function, z = S(x,y), was constructed from the point cloud. Modal reconstruction was chosen due to the high number of failed reconstructions caused by noise in the data input when using zonal methods such as B-splines [7]. In modal reconstruction, the corneal surface is reconstructed by linear expansion, a_j_, of basic polynomial functions (modes), f_j_ (Equation (1)):

z = S (x, y) = ∑ a_j_ f_j_ (x, y), (1)

A widely used criterion in the ophthalmological community to approximate the shape of the corneal surface is to use a model that approximates it to a conical or biconical surface, as proposed by Navarro et al. in 2006 [27]. This biconical surface is expressed as the implicit polynomial equation of a quadric (Equation (2)):

Q(x, y, z) = a_11_ x^2^ + a_12_ x y + a_13_ x z + a_10_ x + a_22_ y^2^ + a_23_ y z + a_20_ y + a_33_ z^2^ + a_30_ z + a_00_ = 0 (2)

It is possible to reconstruct the corneal surface by fitting the coefficients of the polynomial equation, a_ij_, to the input point cloud. Thus, this fitting can be assimilated into a mathematical optimization problem in the following form (Equation (3)):

min Q (a),(3)

This approximation has been successfully used numerous times in biomedical engineering to reconstruct biological surfaces [28,29].

#### 2.3.3. Reconstruction of the Corneal Surface Using NS-MVGA Algorithm CORNEAGA

For solving the optimization problem (Equation (3)) of corneal surface reconstruction, various mathematical programming methods have been used so far, such as matrix methods such as the Moore–Penrose pseudoinverse matrix [30], based on the singular value decomposition of the system matrix, or weighted least squares fitting with trust-region reflective iterative algorithm (TRRA) [31], where the weighting function adjusts to the points in the cloud with higher uncertainty. However, these methods can suffer from instability issues caused by input data or the presence of local minima. A previous study by our research group [32] allowed us to propose, as future work, the use of a metaheuristic search optimization method supported by artificial intelligence. In this study, the application of a solution method through the implementation of a genetic algorithm for mathematical programming has been proposed.

**Figure 2:**
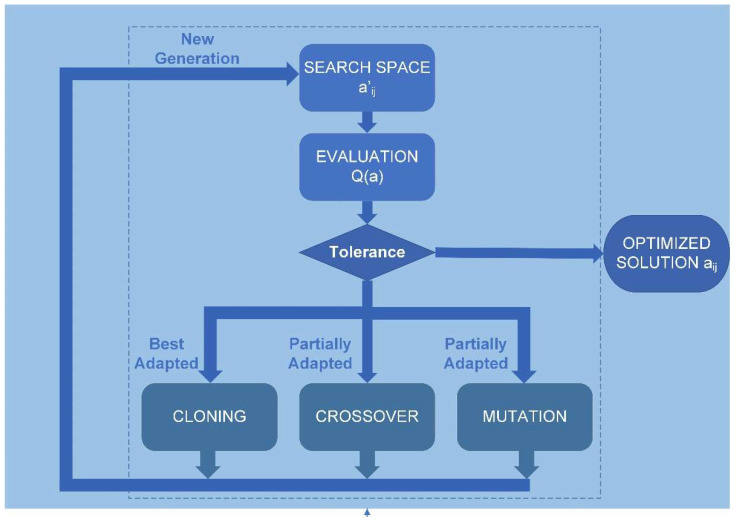
Genetic algorithm flowchart. aij: coefficients of the polynomial of the geometry of a quadric (Equation (2)). Q(a): adaptation function.

The solution to the problem of Equation (3) involves obtaining a solution vector consisting of the parameters a_ij_. Therefore, the genetic algorithm used must have a deterministic multivariable (MVGA) configuration. The genetic algorithm was implemented using Matlab programming, following the scheme in Figure 2, with the script called “*corneaga.m*”. The MVGA was used to minimize the fitness function Q(a_ij_) to obtain the ten adjustment coefficients of the implicit function of the corneal biconical surface (Equation (2)).

The genetic algorithm receives the point cloud data in XYZ Cartesian format after discretization carried out by the “*corneagaimport.m*” script, and the free surface of the cornea determines the problem constraints, which are explicitly introduced through the “*constrains.m*” script (see Figure 2). The constraints are greatly influenced by the input data in other methods [30]. One proposed solution is to introduce a constraint on one of the parameters of Equation ((Equation (2)), such as a_11_ = 1, which provides excellent stability to the solution [27,33]. However, when working with a space of complete solutions, the genetic algorithm is unaffected by such discontinuities as gaps in the input data or other discontinuities. Therefore, the optimal configuration, in this case, is a non-dominant, multi-variable genetic algorithm not constrained by adjustment parameters. It is, therefore, a non-dominated sorting, multi-variable genetic algorithm (NS-MVGA).

A previous study on the application of genetic algorithms in clinical settings [34] allowed our research group to establish the most commonly used parameters in the literature. Then, optimal parameters were selected according to those commonly used in similar studies [35,36], and the genetic algorithm operators used in this study were: (i) a population of 100 individuals for each variable, which implies a global population in each generation of 1000 individuals; (ii) a crossover rate set at 0.8; (iii) a cloning (survival) rate set at 0.2; and (iv) mutation rate set at 0. Additionally, to configure the tolerance parameters of the genetic algorithm, experimentation was carried out with various tests varying these parameters (see Table 1). The optimal result for the tolerance parameters was a value of 10^−50^ for both the objective function and the constraints. With these parameters, optimal values of error and processing time for obtaining the a_ij_ coefficients can be selected.

#### 2.3.4. Obtaining the Reconstructed Surface, Morpho-Geometric Parameters, and Graphical Representation

Once the parameters a_ij_ are obtained, the expression (Equation (2)) is transformed into the classic equation of an ellipsoid (Equation (4)) by means of rotation and orientation using the Matlab function *rot2eul*, with the script called “*elitrans.m*”.

x^2^/a^2^ + y^2^/b^2^ + z^2^/c^2^ = 1, (4)

Here, a, b, and c are the values of the semi-axes of the ellipsoid in the ‘x’, ‘y’, and ‘z’ axes, respectively. This transformation aims to obtain the morpho-geometric parameters used in clinical practice [27]. These parameters are the radii of curvature of the surface in the ‘x’ and ‘y’ axes:

R_x_ = a^2^/c, R_y_ = b^2^/c, (5)

the asphericity in the ‘x’ and ‘y’ axes:

Q_x_ = a^2^/^−2^ − 1, Q_y_ = b^2^/^−2^ − 1, (6)

Subsequently, the reconstructed corneal surface is graphically represented using the “*corneagaplot.m*” script with the *fimplicit3* Matlab function along with the point cloud using the *scatter3* Matlab function. In this script, the surface can be exported in a three-dimensional mesh using three-dimensional Delaunay triangulation [37,38]. The mesh is exported in a stereolithographic format in a .stl file using Matlab’s *stlwrite* function.

#### 2.3.5. Method Validation and Error Calculation

The goodness-of-fit is evaluated within “*corneaga.m*” by calculating each case’s MSE. This is the most commonly used indicator in the literature [30,36]. After obtaining the vector’s ‘a’ from Equation (3), the surface is reconstructed using the formula in Equation (2) and then evaluated at the same nodes where the heights were collected. This gives the vector of adjusted elevations ‘Ẑ’. The MSE is calculated as the average of the squared differences between the adjusted elevations ‘Ẑ’ and the original elevations ‘Z’ (Equation (7)).

MSE = (1/N) ∑ (Ẑ − Z)^2^, (7)

where N is the total number of points.

In Equation (2), some polynomial coefficients may turn null values if there is great uncertainty in the points or a significant lack of data. This implies that some polynomial terms (Equation (2)) can disappear, causing the resulting quadric to not be in the correct order to fit the corneal surface. In other words, a mathematically valid solution is found. However, it is incompatible with the accepted quadric for its approximation to the corneal surface, which, according to a well-known study [27], must be a second-order quadric. This way, a failure quadric number, FQN, can be adopted in the surface reconstruction, which represents the number of times the reconstruction fails out of the total reconstructions performed in the study, a total of 270 corneal surfaces (anterior and posterior surface of each eye). Therefore, it can be expressed as the following equation (Equation (8)):

FQN = ∑_m_ fq_j_, (8)

where fq = 1 if there is a failure in the order number of the quadratic in the reconstruction, and fq = 0 if there is no failure, and m is the counter of reconstructions carried out.

## 3. Results

The complete reconstruction described in Figure 2 was implemented for the experimental input data of each corneal surface for a total of 270 corneal surfaces (anterior and posterior surface of each eye) described in the “data source” section. Additionally, for each of the reconstructions, the implementation of weighted least-squares modal methods, LSQ, and sequential quadratic programming, SQP, was carried out. For each of the reconstructed surfaces, the following were obtained: the coefficients a_ij_ of the adjusted geometric expression of Equation (2); the corneal morpho-geometric parameters R and Q; the mean square error, MSE; the failure rate, FQN; and the graphical representations and reconstructed surface meshes.

Table 2 shows the results of comparing the reconstruction using CORNEAGA and the reconstruction using traditional fitting methods. The average MSE values are shown for each of the studied groups: the control group of healthy patients and the groups of grades according to the Amsler–Krumeich scale. The traditional methods include two methods previously used: LSQ, least squares method with TRRA, and SQP, sequential quadratic programming method.

Table 3 shows the number of reconstruction failures (FQN) for the different methods applied and experimental groups.

**Figure 3:**
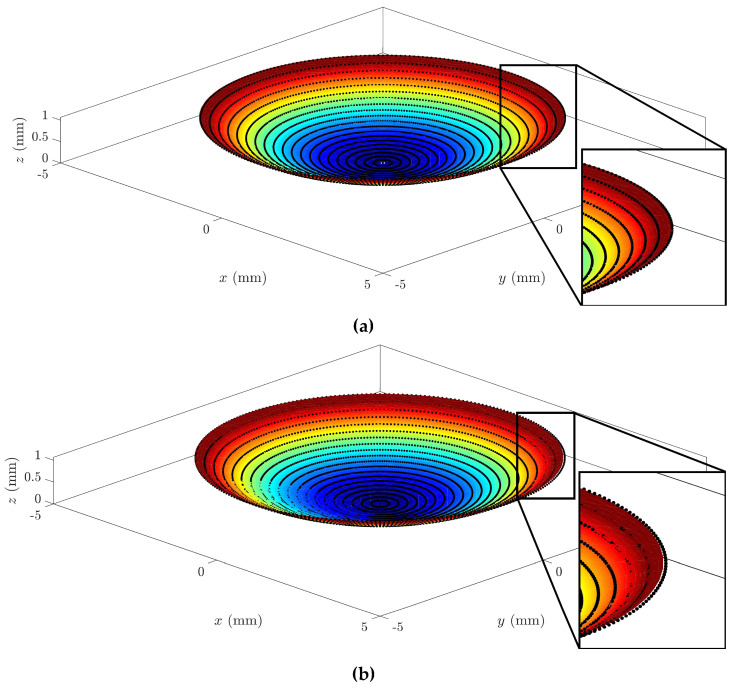
Example of graphical representation of reconstructed corneal surface with enlarged detail for: (**a**) CORNEAGA (top) and (**b**) LSQ (bottom) methods along with the original point cloud in black. (Patient’s characteristics: healthy, age = 29, sex = male, eye = right, central thickness = 512 μm).

**Figure 4:**
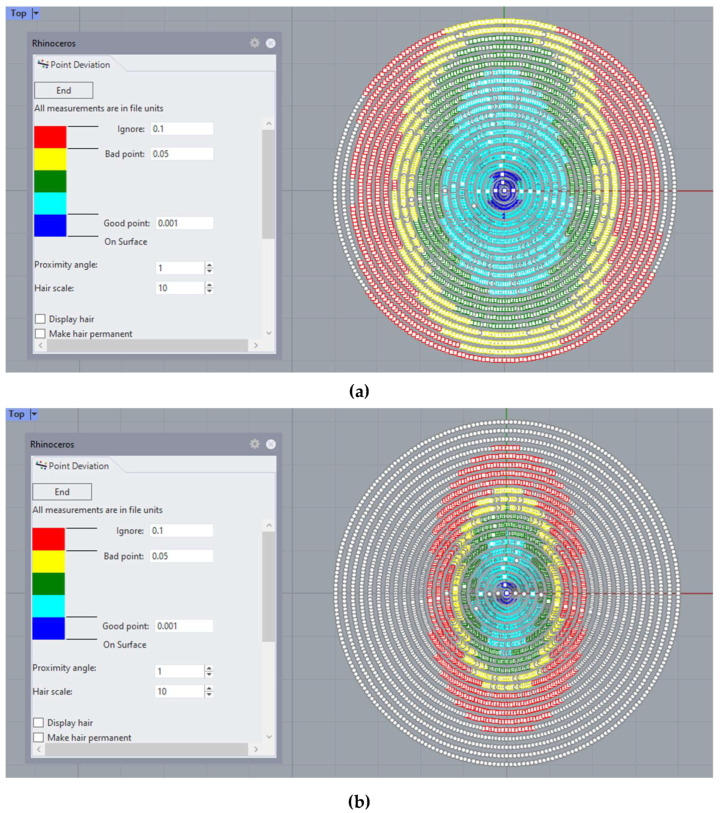
Example of point deviation analysis in Rhinoceros©, where the deviation in elevation between the experimental point cloud and the reconstructed surface is represented using a color scale for: (**a**) the CORNEAGA method (top) and (**b**) LSQ method (bottom). (Patient’s characteristics: healthy, age = 29, sex = male, eye = right, central thickness = 512 μm).

**Figure 5:**
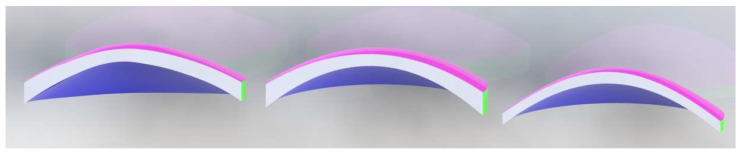
Example of reconstructed cornea in solid, using Solidworks©, using NURBS [1] method (**left**), CORNEAGA method (**center**), and LSQ method (**right**). (Patient’s characteristics: keratoconus, age = 28, sex = female, eye = left, central thickness = 293 μm).

Additionally, the corneal morpho-geometric parameter of asphericity (Equation (6)), related to spherical aberration and refractive index (Q), was calculated, as studied in Navarro et al.’s work [27]. The mean values were calculated for the different methods for the different groups. The results are shown in Table 4:

Another morpho-geometric parameter obtained in the results is the corneal curvature radius of the anterior and posterior corneal surface (ARC/PRC) (Equation (5)) and its relationship with keratoconus pathology, according to a study conducted by Belin et al. [39]. Table 5 shows the values of the curvature radii obtained in the reconstruction of different AK grades using the different methods:

In a graphical analysis, the representation of corneal surfaces for the CORNEAGA and LSQ methods was obtained, where the surface is represented in a colored gradient scale according to the z-axis, along with original tomographic data in the form of black dots. In the following figure (Figure 3), we can see a representation and detail of an example of a corneal surface using both methods:

Additionally, a surface mesh is imported and analyzed using CAD software Rhinoceros, where the deviation in the z-axis (elevation) of points to the original data cloud and their distance to the reconstructed surface can be analyzed (Figure 4):

Finally, using SolidWorks software, the reconstructed anterior and posterior surfaces are imported, and the complete cornea is converted into a closed solid by joining both surfaces. In the following figure (Figure 5), the result of the reconstruction is shown, using CORNEAGA and LSQ methods, compared to the reconstruction using the NURBS zonal method of a complete and sectioned cornea:

## 4. Discussion

Several studies have discussed the morpho-geometric characterization of the human cornea using novel analysis techniques. Modal reconstruction of the corneal surface is part of these fields of study. Considering the limitations of zonal methods, modal methods are a valuable tool for characterizing pathologies such as keratoconus. In present study, the point cloud data of topographic measurements from a Sirius tomographer were used as experimental data, whose use have been previously validated for the characterization of repeatable corneal geometric parameters in healthy eyes [40,41] and those with keratoconus [42,43]. Once the surfaces were reconstructed by the proposed CORNEAGA method and other modal methods, a comparative analysis was performed at the level of goodness of fit by mean squared error, MSE (Equation (7)); failure rate, FQN (Equation (8)), morpho-geometric parameters: curvature radii R (Equation (5)) and asphericity Q (Equation (6)); and, finally, a comparative graphical analysis of the obtained surfaces.

Regarding the mean squared error, MSE, as shown in Table 2, and considering the control group composed of healthy patients, the reconstruction improves by up to 86% using the genetic algorithm CORNEAGA, compared to the least squares method, LSQ. It is observed that as the deformation of the surfaces due to an increasing disease grading rises, this improvement percentage decreases. For this reason, in pathological groups, the improvement percentage reaches, compared to the LSQ method, 58% in AK grade 1 group, 53% in AK grade 2 group, and 58% in AK grades 3 and 4 group. This represents an average improvement of 56% (with a standard deviation of 2.8%) compared to the LSQ method in pathological groups. This is essential for the early detection of this pathology [44].

On the other hand, regarding the SQP method, it is observed that it has the lowest MSE (Table 2) and the highest dispersion among the studied methods, especially in pathological groups. This is due to its inherent instability, which results in faulty artifacts in the reconstruction. Furthermore, as observed in Table 5, the SQP method does not meet the criteria set by Belin et al. in any of the groups.

As for the failure rate, FQN, it can be observed in Table 3 that the new method has a zero-failure reconstruction rate. This is because the new method works with a population of complete solutions, so it always finds a valid solution for the reconstruction. Only a few isolated failures of quadrics have occurred during tests, when the population size in each generation was chosen to be lower than what was established in the “Reconstruction of the Corneal Surface Using NS-MVGA Algorithm-CORNEAGA” section.

Analyzing the corneal asphericity, as shown in Table 4, for the control group consisting of healthy patients, the average asphericity values increase from −0.3114 to −0.3348, which is a significantly higher value than the previous range obtained and indicates a better approximation to the optimal conic constant *Q_opt_* = −0.528 obtained by the authors Navarro et al. in previous publications [27]. As for the pathological groups, it is also observed that asphericity is better approximated to the actual pathological states, whose values are determined to be close to −1 for advanced pathological states [45].

Table 5 shows the relationship between the radii of curvature and the scientific criteria established by Belin et al. [39]. This study suggests a direct relationship between the radii of curvature of the corneal surfaces and the grades of the AK scale (grade 1, ARC/PRC: >7.05/>5.7; grade 2, ARC/PRC: >6.35/>5.15; and grades 3 and 4, ARC/PRC: ~6.15/~4.95). It can be verified that the new CORNEAGA method generates values farther from the limits than the LSQ method, improving the screening of cases. This result is due to the improved precision of the method achieved for this group, which can be observed in Table 2. Similar results are obtained in both methods for the AK grade 2, and the worst results are shown in both methods for the AK grade 3 and AK grade 4.

At a graphical level, Figure 4 shows how the new method fits better to the points in the outer edge zone of the dataset. This border zone represents a discontinuity in the input data. The traditional modal method adapts worse to such discontinuity, as previously confirmed in works by other authors, such as Martinez-Finkelshtein et al. [30]. In Figure 5, it can be seen in the solid section of the entire cornea how the new genetic-algorithm-based method fits much better than the LSQ method to the B-spline NURBS zonal method [25,46], which achieves an MSE of the order of 10^−6^ for this cornea [46], while CORNEAGA achieves an order of 10^−5^ and LSQ only order of 10^−3^. This makes it closer to the most effective zonal method.

## 5. Conclusions

This study aimed to establish a new modal method for corneal surface reconstruction using genetic algorithm fitting. Existing modal methods in the literature have certain limitations that can affect the accuracy of corneal surface reconstruction, especially in cases of discontinuities or incomplete data. Therefore, the objective of this study was to develop a more efficient method that could overcome these limitations.

The results obtained evidence for a reliable new method for corneal surface reconstruction. Additionally, it was found that the new method improves the least squares method by 86%. The results also show an average improvement of 56% (with a standard deviation of 2.8%) compared to the LSQ method in patients with KC, which can allow for early detection of the disease and, therefore, a better prognosis for its progression.

While it does not improve zonal methods in highly deformed surfaces, which achieve an MSE on the order of 10^−6^ [46], the new method represents a significant advancement in modal approximation for cases with incomplete data. The new method has a zero-failure rate in reconstruction due to the inherent goodness of the population with which the algorithm operates.

However, although several sample size calculations were made before the experiment and its size can be considered enough for both healthy and diseased eyes in order to draw general conclusions about the population, it is also true that the AK grade 3 and 4 group of eyes is the most scarce one, and, therefore, it would be advisable to verify the results of this study with a greater sample.

One potential direction for future research could involve reducing the tolerance of the genetic algorithm’s adjustment and enhancing the algorithm’s intergenerational processing capacity through the use of a more advanced computer system. This approach may lead to even greater improvements in the accuracy of the fitting.

In summary, the study has shown that the new method of corneal surface reconstruction is highly effective and significantly improves reconstruction accuracy compared to previous modal methods used in the literature. The new method represents an important advance in modal approximation in cases with incomplete data and has excellent potential for further improvement in the future through the optimization of genetic algorithm adjustments.

## Figures and Tables

**Table 1:** Genetic algorithm parameters adjustment.

Function Tolerance Exponent	Constrain Tolerance Exponent	MSE (10^−3^)	Processing Time (s) ^1^
−2	−2	5.256	32
−10	−10	4.6522	65
−30	−30	0.0804	299
−50	−50	0.0117	299
−100	−1000	0.0804	1972

^1^ Carried out using a Win10 PC with Intel Core i5-7500 CPU @ 3.40 GHz and 16.00 GB RAM.

**Table 2:** MSE: mean and standard deviation values. AK: Amsler–Krumeich scale. LSQ: weighted least squares fitting method. SQP: sequential quadratic programming fitting method.

Method	Control (SD)	AK Grade 1 (SD)	AK Grade 2 (SD)	AK Grade 3 + 4 (SD)
LSQ	8.96 × 10^−3^ (5.25 × 10^−4^)	6.83 × 10^−3^ (1.7 × 10^−3^)	6.03 × 10^−3^ (8.54 × 10^−4^)	6.79 × 10^−3^ (5.06 × 10^−4^)
SQP	1.08 × 10^−2^ (5.78 × 10^−2^)	5.91 × 10^−1^ (1.14 x10^−1^)	7.48 × 10^−1^ (3.40 x10^−1^)	5.08 × 10^−1^ (5.44 x10^−1^)
CORNEAGA	1.24 × 10^−3^ (2.57 × 10^−4^)	2.89 × 10^−3^ (2.74 × 10^−4^)	2.86 × 10^−3^ (1.89 × 10^−4^)	2.88 × 10^−3^ (1.69 × 10^−4^)

**Table 3:** Corneal surface reconstruction failure rate (FQN). AK: Amsler–Krumeich scale. LSQ: least squares fitting method. SQP: sequential quadratic programming fitting method.

Method	Control	AK Grade 1	AK Grade 2	AK Grade 3 + 4
LSQ	5	8	19	11
SQP	25	18	13	2
CORNEAGA	0	0	0	0

**Table 4:** Corneal asphericity parameter Q mean and standard deviation values. AK: Amsler–Krumeich scale. LSQ: least squares fitting method. SQP: sequential quadratic programming fitting method.

METHOD	Control (SD)	AK Grade 1 (SD)	AK Grade 2 (SD)	AK Grade 3 + 4 (SD)
LSQ	−0.3114 (0.1911)	−0.6587 (0.3598)	−0.8206 (0.3837)	−1.1602 (0.5122)
SQP	−0.8216 (0.1636)	−0.8827 (0.2542)	−0.9062 (0.2041)	−1.1602 (0.2214)
CORNEAGA	−0.3348 (0.1637)	−0.6190 (0.2845)	−0.7344 (0.2418)	−0.8202 (0.2241)

**Table 5:** Comparison of anterior radii of curvature (ARC) and posterior radii of curvature (PRC) mean and standard deviation values. AK: Amsler–Krumeich scale. LSQ: least squares fitting method. SQP: sequential quadratic programming fitting method.

METHOD	AK Grade 1 (SD)	AK Grade 2 (SD)	AK Grade 3 + 4 (SD)
LSQ	6.89/5.41 (0.89/1.21)	6.6/5.31 (0.56/1.07)	5.43/4.39 (1.64/0.82)
SQP	4.07/8.45 (5.15/15,38)	2.81/4.04 (2.69/7.16)	1.39/2.71 (2.27/1.63)
CORNEAGA	7.18/5.78 (0.37/0.54)	6.85/5.62 (0.43/0.43)	6.7/5.12 (0.61/0.39)
AK criteria [belin2016] ^1^	>7.05/>5.7	>6.35/>5.15	~6.15/~4.95

^1^ Not a method. Comparison criterion.

## Data Availability

The data presented in this study are available on request from the corresponding author. The data are not publicly available due to privacy reasons.
